# The impact of green HRM practices on green innovative work behaviour: empirical evidence from the hospitality sector of China and Pakistan

**DOI:** 10.1186/s40359-025-02417-5

**Published:** 2025-02-03

**Authors:** Karamat Khan, Eimad Hafeez Gogia, Zhen Shao, Mohd Ziaur Rehman, Assad Ullah

**Affiliations:** 1 School of Digital Commerce, Zhejiang Yuexiu University, Shaoxing, China; 2 School of Economics and Management, Harbin Institute of Technology, Harbin, Heilongjiang China; 3 Department of Finance, College of Business Administration, King Saud University, P.O. Box 71115, Riyadh , 11587 Saudi Arabia; 4 School of Economics & Management, Hainan Normal University, Haikou, China

**Keywords:** Green human resource management, Green innovative work behaviour, Green perceived organizational support, Organizational citizenship behaviour towards the environment, And Green transformational leadership

## Abstract

Amid a global shift towards eco-friendly practices, this study investigates the impact of green human resource management (GHRM) practices on green innovative work behavior (GIWB) in the hospitality sector, focusing on emerging and developing countries, specifically China and Pakistan. The research also explores the mediating roles of green perceived organizational support (GPOS) and organizational citizenship behavior towards the environment (OCBE), as well as the moderating role of green transformational leadership (GTL). This study is grounded in the AMO (ability, motivation, and opportunity) theory and organizational support theory. Data were collected through a questionnaire, yielding 503 valid responses from hotel employees in Beijing, China, and 498 valid responses from Islamabad, Pakistan. Structural Equation Modeling (SEM) was employed to examine and compare the results across both samples. The findings reveal positive and significant relationships between GHRM and GIWB, as well as notable mediation and moderation effects among the specified variables. Interestingly, GTL did not significantly moderate the relationship between GHRM and GIWB in Pakistan’s sample, highlighting a regional contrast. This study contributes to advancing the implementation of GHRM practices to enhance GIWB in the hospitality sector, with insights into the role of GTL across different cultural contexts.

## Introduction

The world stands at a critical juncture, facing an unprecedented global environmental crisis. Climate change, driven by human activities, poses a looming threat to the very ecosystems that sustain life on our planet. Industrialization and the expansion of organizations have significantly contributed to this crisis, spurring debates on corporate responsibility and sustainability. In this era of heightened environmental consciousness, organizations play a pivotal role in either exacerbating or mitigating the challenges posed by climate change and ecological degradation [1]. In response to these challenges, there has been a growing recognition of the role of green human resource management (GHRM) within organizations [2]. GHRM represents a strategic approach to managing an organization’s workforce in a manner that aligns with environmental sustainability principles [3]. It acknowledges that employees are not only the lifeblood of organizations but also influential agents of change in the quest for ecological balance [4]. Studies have shown that organizations with a strong commitment to GHRM tend to have a reduced environmental footprint [5]. These organizations often reap multiple benefits through GHRM practices, including enhanced corporate reputation, increased employee morale and engagement, and improved compliance with environmental regulations [6].

The transformative potential of GHRM lies not only in its ability to reduce the negative environmental impact of organizations but also in its capacity to foster green behaviour among employees [7]. Green innovative work behaviour (GIWB) in this context refers to employees’ dedicated efforts in generating, promoting, and realizing environmentally conscious ideas within the organization [8]. The GIWB extends the concept of innovative work behaviour to incorporate a focus on ecological sustainability. GHRM initiatives create an organizational environment that nurtures a culture of support for green initiatives among employees. When employees perceive GHRM practices as genuine and responsive to environmental concerns, their belief in the organization’s commitment to environmental responsibility strengthens, enhancing green perceived organizational support (GPOS) [9]. Employees who perceive strong organizational support for green initiatives are more likely to internalize the values and objectives of GHRM [10]. This, in turn, fosters their motivation to voluntarily engage in GIWB, encompassing various pro-environmental behaviours beyond their traditional job roles [11].

Organizational citizenship behaviour towards environment (OCBE), represent another channel in the GHRM-GIWB relationship. OCBE represents a range of voluntary, discretionary actions that employees take to promote environmental sustainability within the organization, extending beyond their formal job responsibilities [12]. GHRM practices, such as sustainability training and eco-friendly workplace policies, empower employees and encourage their active involvement in green initiatives [13]. These initiatives signal the organization’s support for employees’ contributions to sustainability efforts and act as source for OCBE. Transformational leadership is another critical driver of organizational success, influencing areas such as strategy, decision-making, and employee behaviour [14]. Green transformational leadership (GTL) has been shown to play a pivotal role in motivating employees to engage in innovative practices, particularly those aligned with sustainability [15]. GTL empowers employees by creating a shared vision of sustainability, fostering a culture of innovation, and aligning organizational objectives with environmental goals [16]. Through such leadership, employees are inspired to embrace and contribute to green initiatives, ultimately enhancing organizational commitment to sustainability [17].

While much of the existing literature on GHRM and GIWB focuses on developed countries, there is a notable lack of research examining how these concepts play out in the hospitality industries of developing and emerging economies [18], such as Pakistan and China. These two countries provide a unique context for studying GHRM and GIWB, as they represent contrasting stages of economic development, regulatory environments, and public awareness of sustainability. China, as the second-largest economy globally, plays a pivotal role in the global hospitality and tourism industry. The market size of China’s hospitality sector is projected to grow significantly, with estimates indicating it will reach USD 86.66 billion in 2024 and is expected to grow at a compound annual growth rate (CAGR) of 8.12% to reach USD 128.04 billion by 2029 [19]. In addition to its robust domestic hospitality sector, China was the most visited country in Asia-pacific region in 2019, attracting approximately 65.7 million international tourists [20]. The hospitality and tourism sector, has been a major contributor to China’s economy, driven by both domestic tourism and international travellers. However, the COVID-19 pandemic severely impacted global tourism, including China, with international arrivals dropping by over 80% in 2020 [21]. Despite the pandemic’s setbacks, China’s tourism sector has shown remarkable resilience, with recovery efforts already underway as domestic travel rebounds and international tourism slowly picks up in 2023 and 2024. In contrast, Pakistan’s hospitality and tourism sector is still developing, with tourism contributing approximately 5.9% to the GDP and creating around 4.2 million jobs in 2022 [22]. Although not as established as China’s, Pakistan’s hotel industry holds considerable potential, particularly with the government’s increasing focus on boosting tourism infrastructure and attracting foreign investment through initiatives like the Pakistan Tourism Strategy 2020–2030 [23]. Exploring these contrasting contexts allows for a comparative analysis of how green HRM practices influence GIWB in different socio-economic and regulatory environments, an area that remains underexplored in the current literature.

In addition, while existing studies have generally looked at the direct relationship between GHRM and GIWB, few have investigated the underlying mechanisms that mediate or moderate this relationship. Specifically, perceived green organizational support (GPOS) and organizational citizenship behavior towards the environment (OCBE) have been identified as potential mediators, but their roles in linking GHRM to GIWB have not been sufficiently examined. In the context of the hospitality sector, where the adoption of green practices is often challenged by resource constraints and diverse employee attitudes, GTL can help amplify the effects of green HRM practices, making them more effective in driving GIWB. This moderating role of leadership has been underexplored in previous research, particularly in hospitality settings, and our study aims to fill this gap by examining how leadership influences the relationship between GHRM and GIWB. By addressing these research gaps, this study provides actionable insights for HR practitioners, policymakers, and industry stakeholders, helping them design and implement effective green HRM strategies that align with their country’s unique socio-economic and regulatory environment. Furthermore, it contributes to the growing body of knowledge on sustainability in the hospitality sector, particularly in developing and emerging economies like Pakistan and China, which are facing distinct environmental challenges and opportunities.

 The subsequent sections of this study are structured as follows: Section "Theoretical Foundation, Literature Review, and Hypothesis Development" presents an extensive literature review, establishes the theoretical framework, and outlines the development of our hypotheses. Section "Materials and Methods", describes the methodology and measures adopted for this research. Section "Main Results: Structural Equation Modelling" presents the main findings of the study. Lastly, Section "Conclusion" concludes by discussing the results and delving into their managerial and theoretical implications.

## Theoretical foundation, literature review, and hypothesis development

### Theoretical foundation

This study draws upon the AMO (ability-motivation-opportunity) theory and organizational support theory. The AMO theory, rooted in organizational psychology and management field, consists of three key components: ability, motivation, and opportunity [24]. Ability relates to an individual’s skillset and knowledge relevant to their role. Motivation pertains to the psychological drive and enthusiasm to excel, fuelled by both intrinsic and extrinsic factors. Opportunity encompasses the external conditions and resources provided by the organization that enable individuals to apply their abilities and channel their motivation effectively. This theory highlights how the interaction of these elements’ shapes both individual and organizational performance, emphasizing the need for alignment and support in each facet to achieve optimal outcomes [25]. The present study is centred around the AMO theory and its application to GHRM practices and their resulting outcomes. In this context, abilities can be assessed and cultivated through the implementation of GHRM practices [26]. These abilities empower employees and staff to transition from ability to motivation, leading to the display of organizational citizenship behaviour in support of environmental initiatives. When employees are motivated, they are more inclined to engage in citizenship behaviour directed at the environment, and they are more likely to contribute to green outcomes, such as green work behaviour [27]. Regarding opportunity, organizations can create an environment that fosters opportunities for employees to exhibit green creativity and innovation [28]. AMO theory’s relevance extends to the green transformational leadership (GTL) as GTL plays critical role in optimizing the interaction between ability, motivation, and opportunity in the context of environmental sustainability [29]. Organizational support theory (OST) is another framework in organizational psychology that explores how employees perceive the level of support provided by their organization and how it affects employee attitudes and behaviours [30]. In the context of green initiatives, OST is relevant because it helps us understand how employees’ perceptions of support for environmentally conscious practices can influence their engagement in GIWB [31].

### Green Human Resource Management (GHRM) and Green Innovative Work Behaviour (GIWB)

Numerous studies have identified the favourable relationship between green human resource management (GHRM) practices and green innovative work behaviour (GIWB). These practices can be effectively understood through the lens of the ability-motivation-opportunity (AMO) theory, which explains how organizational practices can shape employee behaviours by enhancing their ability, boosting their motivation, and creating opportunities to engage in desired actions. For instance, GHRM practices such as green coaching and training directly enhance employees’ abilities to engage in innovative environmental initiatives, equipping them with the necessary skills and knowledge to identify and implement innovative green solutions [32]. This aligns with the ability component of the AMO framework, which posits that employees must be capable of performing the tasks required for green innovation. Additionally, GHRM practices such as green rewards and performance appraisals also play a critical role in fostering GIWB. Rewards and recognition tied to environmental objectives motivate employees to align their behaviour with the organization’s green goals, driving employee engagement and commitment to green innovation [33, 34]. This corresponds to the motivation element of the AMO theory, as employees are incentivized to perform better when their efforts are acknowledged and rewarded.

Furthermore, GHRM practices create an enabling environment for employees to participate in green initiatives by fostering a culture of innovation and sustainability. Employees’ attributions regarding the reasons behind HR practices significantly influence their attitudes and behaviours, which, in turn, impact organizational outcomes [35]. Research suggests that green HRM practices encourage employees to take discretionary actions, such as proposing and implementing sustainable innovations [36]. This reflects the opportunity dimension of the AMO framework, emphasizing the importance of providing employees with platforms and conditions that allow them to act on their innovative potential. In summary, GHRM practices significantly contribute to GIWB by enhancing employees’ ability to innovate, motivating them through rewards and recognition, and providing opportunities to act [32–36].

- H_1_: Green human resource management practices are significantly related to green innovative work behaviour.

### Mediating role of Green Perceived Organizational Support (GPOS)

The primary objective of green human resource management (GHRM) is to align human resource practices with environmental sustainability goals, ultimately fostering positive and green organizational outcomes. Research suggests that the activities and practices associated with GHRM are designed to be environmentally conscious, contributing to ecological stability [13]. By emphasizing eco-friendly processes, GHRM transforms conventional HRM practices, leading to the development of a green workforce that is committed to sustainable principles [28]. A key component of GHRM is the active engagement of upper management in promoting environmentally friendly processes and encouraging employees to participate in initiatives aimed at reducing environmental pollution. The success of GHRM practices is closely linked to how companies prioritize employee concerns within the framework of environmental sustainability [10].

In line with this, research indicates that when employees perceive organizational support, they are more likely to reciprocate through positive organizational outcomes such as increased commitment, job satisfaction, and discretionary behaviors [30, 37]. Furthermore, employees have the potential to enhance their contributions to organizations by acquiring the necessary expertise and skills to foster innovative work behaviors, particularly in environmentally sustainable initiatives [38]. Previous studies have corroborated a positive relationship between employees’ perception of organizational support (POS) and their engagement in innovative work behaviour. These studies suggest that when employees feel supported by their organizations, it fosters a sense of trust in the organizational environment. This trust, in turn, amplifies their confidence that their contributions will be duly acknowledged, while simultaneously diminishing perceived risks linked to undertaking innovative initiatives [39, 40]. Studies also suggest that GHRM practices exert influence not only through direct channels on employees’ environmentally friendly behaviours but also operate through various psychological and social processes [33, 41]. This study proposes that green perceived organizational support (GPOS) functions as a mediating mechanism, connecting GHRM practices to the green innovative work behaviour of employees.

- H_2_: Green perceived organizational support significantly mediates the relationship between green human resource management practices and green innovative work behaviour.

### Mediating role of Organizational Citizenship Behaviour towards Environment (OCBE)

Organizational citizenship behaviour (OCB) refers to voluntary and discretionary actions employees take that go beyond the requirements of their formal job duties. GHRM practices, such as green promotion, green evaluation, and green rewards and compensation, provide tangible recognition of employees’ environmental efforts, motivating them to exhibit voluntary green behaviours [42]. This creates a positive feedback loop, where employees feel appreciated and valued for their environmental contributions, which enhances their sense of obligation to engage in green behaviours. The perception of being valued by the organization increases employees’ motivation to act in environmentally responsible ways, ultimately contributing to greater OCBE and environmental performance [4, 41]. From the perspective of AMO theory, GHRM practices enhance employees’ ability, motivation, and opportunity, thus fostering OCBE and its downstream effect on green innovative work behaviour (GIWB). By providing employees with the necessary skills and knowledge, GHRM practices ensure that they are equipped to undertake environmental initiatives [43]. GHRM practices that explicitly value and reward green behaviours motivate employees to exceed job expectations and actively contribute to OCBE [42]. The perception of being recognized for their green efforts strengthens employees’ intrinsic and extrinsic motivation, fostering a commitment to environmental responsibility [44]. This aligns with the motivation component of AMO theory, as employees are more likely to engage in behaviours that are both personally fulfilling and rewarded by the organization. This creates a conducive environment for employees to manifest their green innovative work behaviours [45].

- H_3_: Organizational citizenship behaviour towards environment significantly mediates the relationship between green human resource management practices and green innovative work behaviour.

### Moderating role of green transformational leadership

Transformational leadership, which emphasizes charisma, intellectual stimulation, individualized consideration, and inspirational motivation, plays a key role in driving sustainable practices by inspiring employees to embrace environmental responsibility and innovation [46, 47]. Leaders with this style can align green human resource management (GHRM) practices with broader sustainability goals, fostering a culture of environmental responsibility [17]. Through the lens of AMO theory, transformational leadership enhances employees’ ability by providing intellectual stimulation and training, strengthens motivation by fostering a sense of purpose and recognition, and creates opportunities for employees to contribute to green innovation by addressing their individual needs and aspirations [29, 48]. By creating a supportive, inclusive environment, transformational leaders not only align with GHRM objectives but also promote collective commitment to green innovation [28].

- H_4_: Green transformational leadership significantly moderates the relationship between green human resource management practices and green innovative work behaviour.

**Fig. 1:**
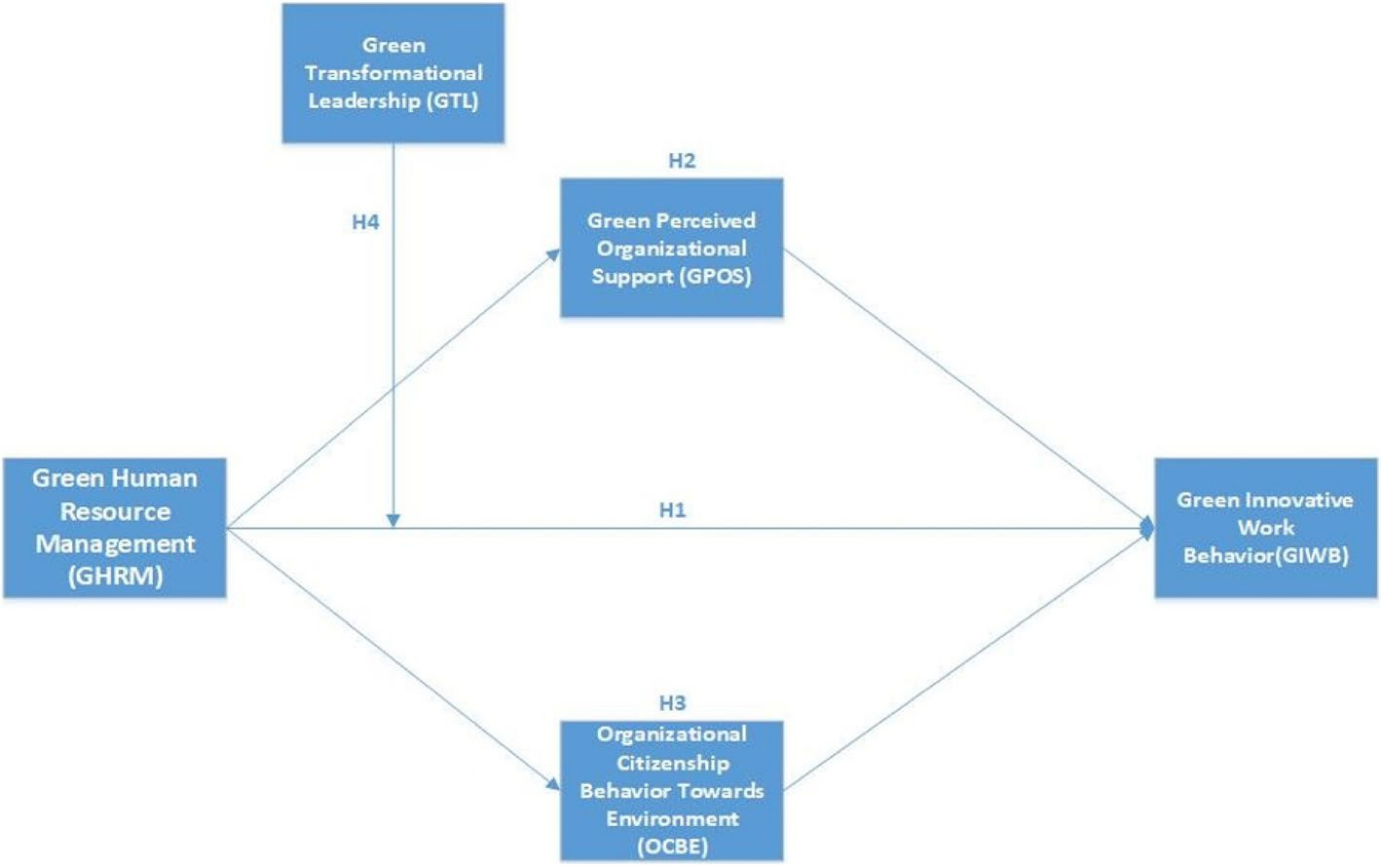
Conceptual framework

Building on the previous research, this study explores the impact of green human resource management (GHRM) on green innovative work behaviour (GIWB), introducing organizational citizenship behaviour towards environment (OCBE) and green perceived organizational support (GPOS) as mediating factors. The unique aspect of this framework lies in investigating both GPOS and OCBE as mediators simultaneously. Additionally, the framework incorporates the moderating role of green transformational leadership (GTL) to enhance understanding. Figure 1 illustrates the proposed conceptual framework of the study.

## Materials and methods

### Methodology

This study utilizes a cross-sectional design to investigate the relationship between independent and dependent variables. Primary data are obtained through questionnaires designed for each variable. A 5-point Likert scale was utilized to construct instruments for each variable. More than 550 questionnaires were initially distributed in hotels located in the capital cities of China (Beijing) and Pakistan (Islamabad). Following data collection, responses were carefully reviewed, with incomplete or missing data removed during the cleaning process. This resulted in a final valid sample size of 503 responses from Beijing and 498 responses from Islamabad. A convenient sampling method was employed for data collection. The instruments used in the study were adapted from various sources. The questionnaire incorporates demographic variables such as age, gender, work experience, educational qualification, etc. Additionally, it covers other variables, including green human resource management (GHRM), green innovative work behaviour, organizational citizenship behaviour, green perceived organizational support, and green transformational leadership. The study includes responses from employees across all levels (executive, middle, and operational) working in hotels in Islamabad and Beijing. Exploratory factor analysis and structural equation modelling (SEM) were performed using SMART PLS. Structural equation modelling (SEM) is a powerful statistical method used to examine the relationships between latent variables and their constructs, helping validate theoretical models. It enables researchers to explore and confirm how multiple variables are interconnected. SEM is particularly useful because it minimizes errors in the model while investigating relationships among latent constructs [2]. There are two main approaches to SEM: covariance-based (CB-SEM) and variance-based (PLS-SEM). Although CB-SEM has traditionally been the more common technique, the use of PLS-SEM has grown significantly in recent years due to its flexibility and advantages. For this study, PLS-SEM was used to test the proposed hypotheses using the Smart PLS software. PLS-SEM was chosen for several reasons [49, 50]. It is considered more suitable for complex models, especially those involving moderation or mediation effects. Additionally, it is simpler to use and does not require the data to meet the assumption of normality, making it a robust choice for structural analysis. The method has gained popularity in recent studies for its ease of application and reliability in analysing relationships between variables.

### Measurements

The study employs instruments adapted from various sources to operationalize different constructs. Table 1 presents an overview of these constructs, including the number of items and their respective sources. Green human resource management practices consist of six items adapted from Dumont et al. (2017) [41]. Green innovative work behaviour is assessed through six items adapted from Scott & Bruce (1994) [51]. Organizational citizenship behaviour towards the environment is measured using three items adapted from Boiral and Paille (2012) [52]. Green perceived organizational support is evaluated through a seven-item scale developed by Eisenberger et al. (1986) [37]. Green transformational leadership is gauged using a six-item scale adapted from Chen & Chang (2013) [53].

**Table 1:** Measurements

Constructs	Items	Source
Green Human Resource Management Practices	GHRM1, GHRM2, GHRM3, GHRM4, GHRM5, GHRM6	(Dumont et al., 2017) [41]
Green Innovative Work Behavior	GIWB1, GIWB2, GIWB3, GIWB4, GIWB5, GIWB6	(Scott & Bruce, 1994) [51]
Organizational Citizenship Behavior towards Environment	OCBE1, OCBE2, OCBE3	(Boiral & Paillé, 2012) [52]
Perceived Green organizational support	GPOS1, GPOS2, GPOS3, GPOS4, GPOS5, GPOS6, GPOS7	(Eisenberger et al., 1986) [37]
Green Transformational Leadership	GTL1, GTL2, GTL3, GTL4, GTL5, GTL6	(Chen & Chang, 2013) [53]

Source: Developed by the author

### Demographics

In Table 2, a comparative analysis of demographic trends in the hotel industry of China and Pakistan is presented. The sample size follows the rule of thumb, which recommends that sample size should exceed the total number of items used in the questionnaire [54]. Nearly 500 responses were collected from employees in hotels located in Beijing and Islamabad. To ensure both accuracy and a higher number of responses, this research opted for a convenience sampling method and shorter questionnaires. This approach was chosen to mitigate survey fatigue and accommodate the time constraints faced by respondents [55].

In China, the gender distribution indicates more female respondents (57.1%) than male respondents (42.9%). Conversely, in Pakistan, male respondents constitute a higher percentage (69.1%) compared to female respondents (30.9%). Examining age groups, the majority of employees in Beijing fall within the 31–35 age range (30.8%), while the lowest representation is observed in the 46 and above age group (4.4%). In Pakistan, the largest proportion of employees is aged 26–30 (33.1%), and the lowest representation is found in the 46 and above age group (1.8%). Regarding work experience, in China, the highest percentage of respondents have 6–10 years of experience (32.6%), while the least experienced employees have 21 years and above (2.4%). In Pakistan, the majority of hotel employees fall within the 6–10 years’ experience category (40.2%), with the least experienced employees in the 21 years and above group (0.2%). Educational qualifications reveal that in China, a significant portion of employees hold a Master’s degree and above (34.2%), followed by those with Bachelor’s degree (30.4%). In Pakistan, the majority of employees possess Bachelor’s degrees (40.2%) or Intermediate degrees (35.3%).

**Table 2:** Demographics

China			Pakistan		
**Gender**	**Frequency**	**Percentage**	**Gender**	**Frequency**	**Percentage**
Male	216	42.9	Male	344	69.1
Female	287	57.1	Female	154	30.9
**AGE**			**AGE**		
20–25	58	11.5	20–25	54	10.8
26–30	119	23.7	26–30	165	33.1
31–35	155	30.8	31–35	141	28.3
36–40	108	21.5	36–40	114	22.9
41–45	41	8.2	41–45	15	3.0
46-above	22	4.4	46-above	9	1.8
**Experience**			**Experience**		
0–5	106	21.1	0–5	141	28.3
6–10	164	32.6	6–10	200	40.2
11–15	160	31.8	11–15	133	26.7
16–20	61	12.1	16–20	23	4.6
21- above	12	2.4	21- above	1	0.2
**Education**			**Education**		
High school	96	19.1	High school	176	35.3
Bachelor’s degree	153	30.4	Bachelor’s	200	40.2
Master’s degree and above	172	34.2	Master’s and above	86	17.3
Others	82	16.3	Others	36	7.2

**Source**: Estimated by the author

## Main results: structural equation modelling

Next, the authors employed structural equation modelling (SEM) to assess the reliability, validity, and study hypothesis. The first part of SEM comprises the measurement model, where the analysis checks the reliability and validity of the items corresponding to the constructs utilized in our research. The robustness of the construct is thoroughly examined through various measures, including factor loading, Cronbach’s alpha value, composite reliability, and average variance extracted (AVE). These metrics collectively assess the quality of the constructs in our study. Furthermore, we evaluated the discriminant validity to ensure differences or discrimination among the variables, thereby confirming that the measured constructs are distinct. The evaluation of discriminant validity included the use of the Heterotrait-Monotrait Ratio (HTMT) table, which quantitatively examines the distinctiveness of construct. Furthermore, the relationships between independent and dependent variables, as well as the mediation and moderation effects, were examined using the bootstrapping method in SMART-PLS. This rigorous analysis enhances the reliability of our findings and provides a robust foundation for drawing meaningful conclusions from the structural equation modelling analysis.

Table 3 presents the results of the factor loading for all items related to the variables under investigation in this study. The data from China indicates that all items exhibit factor loadings above 0.7, signifying that the data is well-suited for subsequent analysis [56]. Moreover, the Cronbach’s alpha values for all variables surpass 0.7, indicating high reliability, and the average variance values exceed 0.5. The factor loading and reliability of data from Pakistan shows that all values are above 0.7 except some items’ values, which are above 0.6. Some scholars suggest that standardized factor loading with a threshold of 0.6 and above is also suitable [57]. Additionally, Table 3 reports AVE values ranging between 0.5 and 0.7, suggesting that all items are reliable and suitable for further analysis [58].

**Table 3:** Factor loading and reliability

China						Pakistan					
Construct	Items	Factor Loading	Cronbach’s Alpha	CR	AVE	Construct	Items	Factor Loading	Cronbach’s Alpha	CR	AVE
	GHRM1	0.754				GHRM1		0.660			
	GHRM2	0.811				GHRM2		0.695			
GHRM	GHRM3	0.793	0.921	0.921	0.661	GHRM3		0.896	0.944	0.941	0.730
	GHRM4	0.818				GHRM4		0.938			
	GHRM5	0.876				GHRM5		0.987			
	GHRM6	0.822				GHRM6		0.898			
	GIWB1	0.781				GIWB1		0.856			
	GIWB2	0.726				GIWB2		0.860			
	GIWB3	0.766	0.885	0.884	0.561	GIWB3		0.879			
GIWB	GIWB4	0.725				GIWB4		0.758	0.931	0.930	0.691
	GIWB5	0.753				GIWB5		0.842			
	GIWB6	0.740				GIWB6		0.785			
	GPOS1	0.767				GPOS1		0.734			
	GPOS2	0.779				GPOS2		0.816			
	GPOS3	0.792				GPOS3		0.755			
GPOS	GPOS4	0.836	0.922	0.922	0.629	GPOS4		0.843	0.901	0.901	0.565
	GPOS5	0.805				GPOS5		0.706			
	GPOS6	0.805				GPOS6		0.720			
	GPOS7	0.766				GPOS7		0.676			
	GTL1	0.756				GTL1		0.841			
	GTL2	0.778				GTL2		0.755			
GTL	GTL3	0.733	0.890	0.890	0.576	GTL3		0.755	0.931	0.930	0.694
	GTL4	0.732				GTL4		0.946			
	GTL5	0.830				GTL5		0.963			
	GTL6	0.718				GTL6		0.701			
	OCBE1	0.739				OCBE1		0.694			
OCBE	OCBE2	0.756	0.787	0.787	0.551	OCBE2		0.812	0.769	0.771	0.530
	OCBE3	0.732				OCBE3		0.671			

**Source**: Estimated by the author

The heterotrait-monotrait ratio test (HTMT) was utilized to evaluate discriminant validity in this study, a statistical technique commonly employed in structural equation modelling (SEM) to assess the distinctiveness of constructs. A threshold value, often set at 0.85 or 0.90, is typically used to determine acceptable discriminant validity, with values below this threshold indicating sufficient differentiation between constructs [59]. In the present study, a threshold of 0.85 was selected. In Table 4, all HTMT values for the measured constructs in both the Chinese and Pakistani datasets were below this threshold, which shows that constructs are distinct from each other.

**Table 4:** Discriminant validity

China							Pakistan						
**Constructs**	1	2	3	4	5	6	**Constructs**	1	2	3	4	5	6
GHRM							GHRM						
GIWB	0.750						GIWB	0.235					
GPOS	0.738	0.769					GPOS	0.265	0.540				
GTL	0.678	0.748	0.664				GTL	0.164	0.365	0.556			
OCB	0.607	0.725	0.647	0.604			OCB	0.180	0.454	0.555	0.388		
GTL x GHRM	0.604	0.491	0.649	0.631	0.547		GTL x GHRM	0.064	0.099	0.235	0.095	0.113	

**Source**: Estimated by the author

**Fig. 2:**
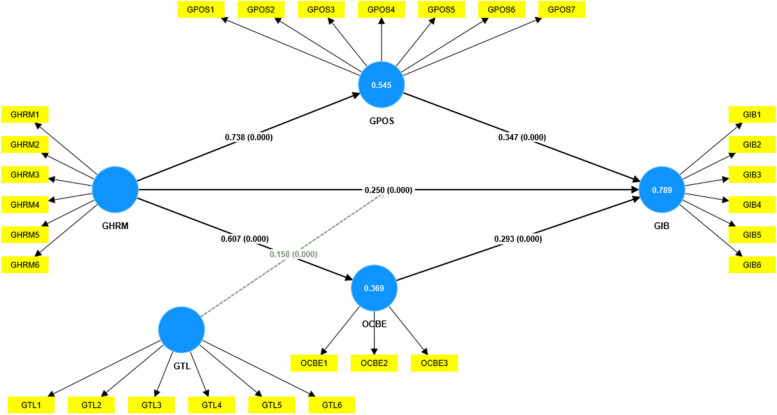
Path coefficient and factor loading (China). Source: Estimated by the author

This study aimed to explore the nexus between GHRM and GIWB, while also investigating potential mediating and moderating factors. Table 5; Fig. 2 report the results obtained from an empirical investigation carried out in the Chinese context. Hypothesis 1 (H_1_) proposes a direct relationship between GHRM and GIWB, and the results indicate strong support for this hypothesis. The standard beta of 0.250, t-Stats of 3.807, and a *p*-Value of < 0.05 confirm a positive and statistically significant impact of GHRM on GIWB. The R-squared value provides insights into the goodness of fit of the model. It assesses how well the independent variables in the model explain the variability in the dependent variable, while also considering the number of predictors. In our regression analysis, the adjusted R-squared value of 0.787 indicates a strong goodness of fit, suggesting that approximately 78.7% of the variability in the dependent variable is explained by the independent variables. The Q^2^ value of 0.515 further supports the model’s predictive ability, emphasizing its reliability in capturing and explaining the observed variance in the dependent variable. Next, hypothesis 2 (H_2_) introduces green perceived organizational support (GPOS) as a mediator in the GHRM-GIWB relationship. The results, with a standard beta of 0.256, t-Stats of 4.925, and a *p*-Value of < 0.05, support the notion that GPOS significantly mediates the relationship between GHRM and GIWB. Hypothesis 3 (H_3_) explores the mediation effect of organizational citizenship behaviour towards the environment (OCBE) in the GHRM-GIWB relationship. The results, with a standard beta of 0.178, t-Stats of 3.882, and a highly significant *p*-Value of < 0.05, provide evidence supporting the positive impact of OCBE in mediating the relationship between GHRM and GIWB. Finally, Hypothesis 4 (H_4_) introduces green transformational leadership (GTL) as a moderator in the GHRM-GIWB relationship. The results, with a standard beta of 0.158, t-Stats of 3.550, and a *p*-Value of < 0.05, indicate that GTL significantly moderates the relationship between GHRM and GIWB, suggesting that the presence of GTL amplifies the positive influence of GHRM on GIWB.

**Table 5:** Main results

China						
Hypothesis	Standard Beta	t-Stats	*p*-Value	LLCI	ULCI	Results
GHRM → GIWB (**H**_**1**_)	0.250	3.807	0.000	0.123	0.377	Supported
GHRM→GPOS→ GIWB (**H**_**2**_)	0.256	4.925	0.000	0.154	0.358	Supported
GHRM→OCBE→GIWB (**H**_**3**_)	0.178	3.882	0.000	0.102	0.28	Supported
GTL×GHRM→GIWB (**H**_**4**_)	0.158	3.550	0.000	0.068	0.243	Supported
**R**^**2**^	0.787					
**Q**^**2**^	0.515					

**Source**: Estimated by the author

**Fig. 3:**
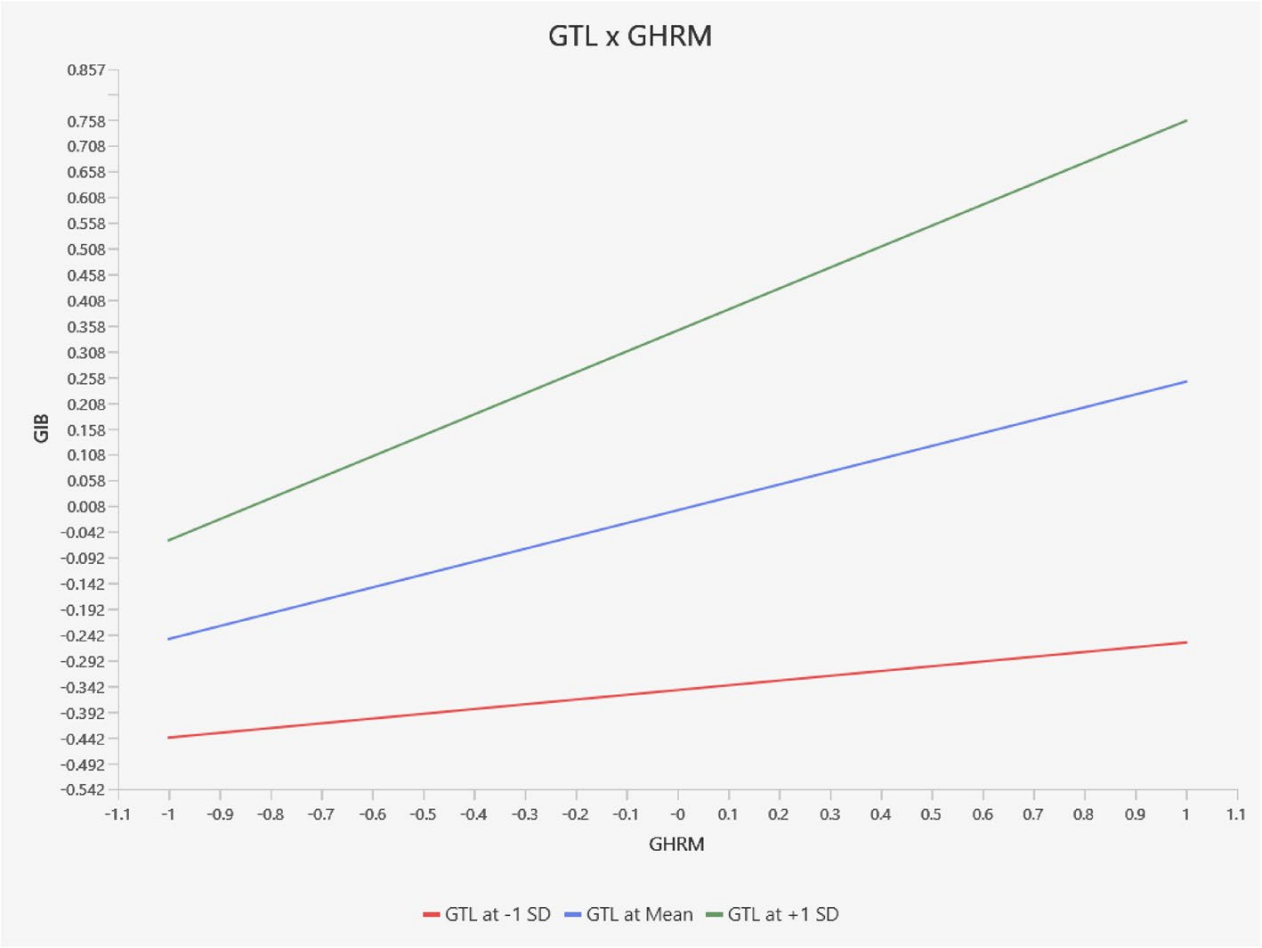
Slope analysis (China). Source: Estimated by the author

The slope analysis conducted using SMART-PLS provides further insights into the moderating effect of GTL on the relationship between GHRM and GIWB. Figure 3 illustrates this relationship through three lines: the middle line represents the relationship at an average level of GTL, while the other two lines depict the relationship at higher (mean value of GTL plus one standard deviation) and lower (mean value of GTL minus one standard deviation) levels of GTL. The graph reveals that the relationship between GHRM and GIWB strengthens as the level of GTL increases. At high levels of GTL, the slope is steeper, indicating a stronger positive relationship between GHRM and GIWB. This suggests that leaders who actively promote environmental goals enhance the effectiveness of GHRM practices in driving innovative work behaviour. In conclusion, the findings from this analysis provide robust empirical support for the direct relationship between GHRM and GIWB, as well as the mediating role of GPOS and OCBE, and the moderating effect of GTL in the Chinese hospitality context. These results contribute valuable insights into the dynamics of green management practices and their impact on fostering green innovative work behaviour.

**Fig. 4:**
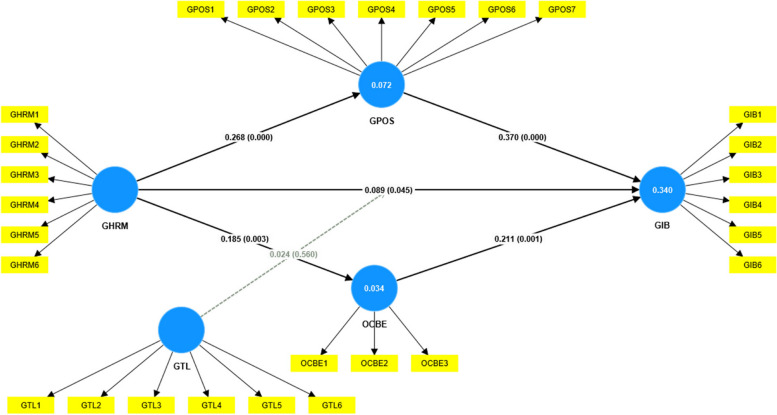
Path coefficient and factor loading (Pakistan). Source: Estimated by the author

Next, the research delved into examining the relationship between GHRM and GIWB, while also exploring the moderating and mediating relationships within this nexus, in the context of Pakistani hotel industry. The detailed findings of this investigation are provided in Table 6; Fig. 4. Hypothesis 1 (H_1_), which posits a direct relationship between GHRM and GIWB, is supported, with a significant standard beta coefficient of 0.089, a t-Stats value of 2.001, and a *p*-value of < 0.05. This suggests that GHRM has a positive and statistically significant impact on GIWB. In our regression analysis, the coefficient of determination (R-squared) was found to be 0.334. This indicates that approximately 33.4% of the variance in the dependent variable GIWB can be explained by the independent variable GHRM. Furthermore, hypothesis 2 (H_2_), introducing GPOS as a mediator, is also supported. The significant standard beta coefficient of 0.099, t-Stats of 3.702, and a *p*-value of < 0.05 indicate a mediated relationship between GHRM and GIWB. Similarly, hypothesis 3 (H_3_), incorporating OCBE as a mediator, is supported, indicating a positive and significant impact on GIWB. However, this relationship is significant at a level of *p* < 0.05 level of significance.

**Table 6:** Main results

Pakistan						
Hypothesis	Standard Beta	t-Stats	*p*-Value	LLCI	ULCI	Results
GHRM → GIWB (**H**_**1**_)	0.089	2.001	0.045	0.005	0.178	Supported
GHRM→GPOS→ GIWB (**H**_**2**_)	0.099	3.702	0.000	0.053	0.156	Supported
GHRM→OCBE→GIWB (**H**_**3**_)	0.039	2.093	0.036	0.009	0.081	Supported
GTL×GHRM→GIWB (**H**_**4**_)	0.024	0.583	0.560	−0.054	0.103	Not Supported
**R**^**2**^	0.334					
**Q**^**2**^	0.086					

**Source**: Estimated by the author

**Fig. 5:**
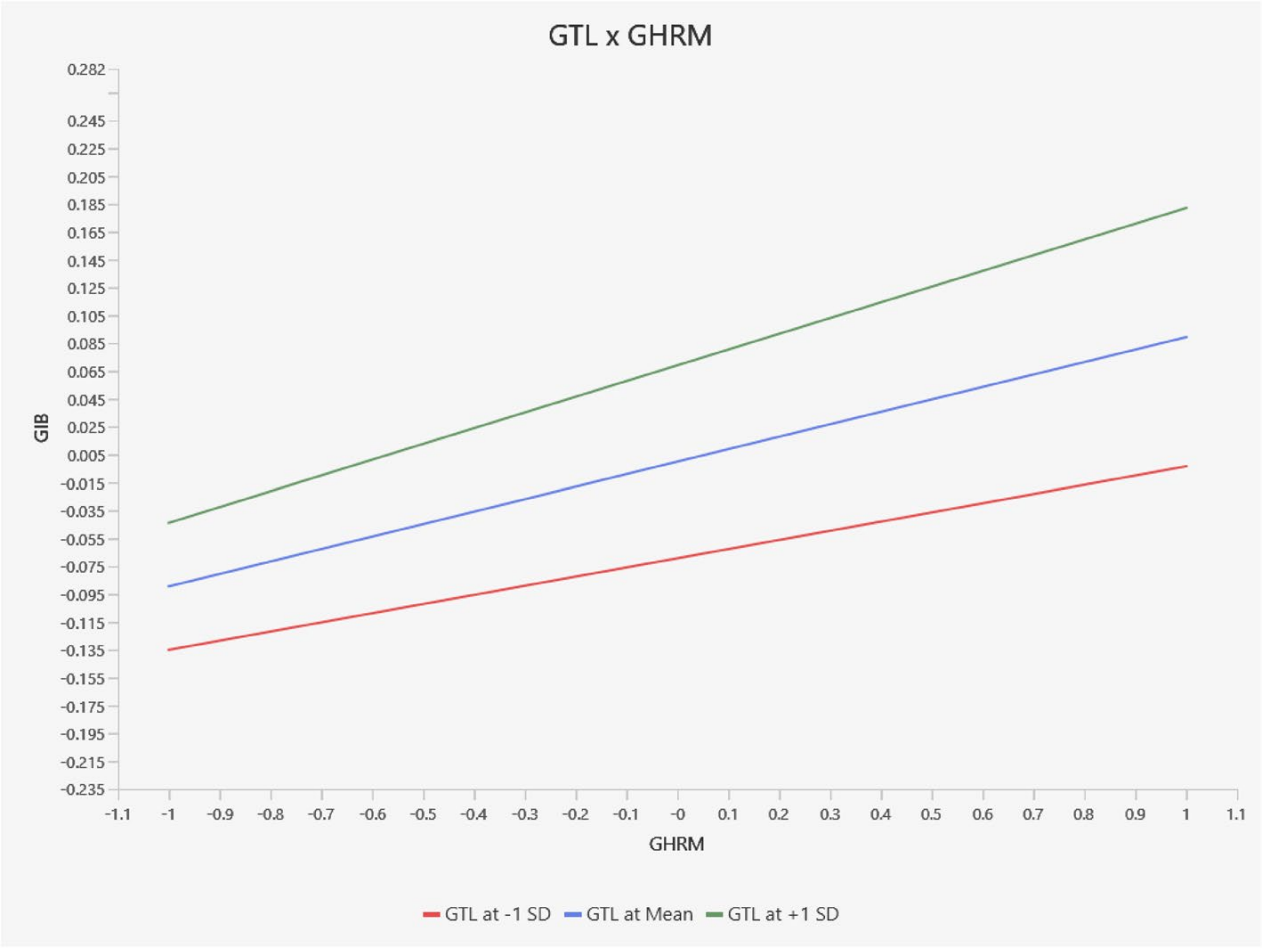
Slope analysis (Pakistan). Source: Estimated by the author

Notably, hypothesis 4 (H_4_), which involves the interaction between GTL and GHRM, is not supported. The slope analysis for Pakistan in Fig. 5 indicates that, although the slopes remain positive, they are relatively flatter. This suggests that variations in GTL do not significantly enhance or diminish the influence of GHRM on GIWB in the context of Pakistan, even though the positive relationship between GHRM and GIWB persists.

Finally, this study estimated the differences between groups through a comprehensive multiple-group analysis (MGA) to determine potential differences in the relationships within our proposed model across two different contexts: China and Pakistan. MGA is a valuable statistical technique that allows us to investigate whether the structural paths and coefficients differ significantly between these groups [58], shedding light on the contextual variations that may influence the relationships under investigation. Table 7 presents the path coefficient differences and associated *p*-values for critical relationships within the model, comparing China and Pakistan. The relationship between GHRM and GIWB exhibits a significant difference (0.161, *p* = 0.039), emphasizing that the impact of GHRM on GIWB significantly varies between the two countries. When considering the mediating effect of GPOS, the path coefficient difference is also significant (0.157, *p* = 0.009), indicating that the indirect pathway through GPOS differs notably between China and Pakistan. In the case of the mediated relationship involving OCB, the path coefficient difference is highly significant (0.139, *p* = 0.001), underscoring considerable variability in the mediating effect of OCBE across the two countries. The interaction term featuring GTL moderating the GHRM-GIWB relationship also exhibits a significant difference (0.134, *p* = 0.025), highlighting that the moderating effect of GTL varies significantly between China and Pakistan. These results collectively indicate that the relationships within the proposed model significantly differ between China and Pakistan. The variations in path coefficients suggest that the impact of GHRM on GIWB, as well as the mediating role of GPOS and OCBE, and the moderating effect of GTL, are influenced by contextual factors inherent to each country. These findings underscore the importance of considering cultural, organizational, and contextual complexities when formulating and implementing green management practices, providing valuable insights for practitioners and policymakers seeking to enhance sustainable practices in both the Chinese and Pakistani organizational contexts.

**Table 7:** Path coefficient difference (China-Pakistan)

Bootstrap (MGA)	Difference (Group China - Group Pakistan)	2-tailed (Group_China vs. Group_Pakistan) *p* value
GHRM → GIWB	0.161	0.039
GHRM→GPOS→ GIWB	0.157	0.009
GHRM→OCBE→GIWB	0.139	0.001
GTL×GHRM→GIWB	0.134	0.025

**Source**: Estimated by the author

## Conclusion

The aim of this research was to examine how green human resource management influences green innovative work behaviour within the hospitality sector of China and Pakistan. The study sought to investigate this connection by considering the mediating roles of green perceived organizational behaviour and organizational citizenship behaviour towards the environment. Additionally, our study aimed to evaluate the moderating impact of green transformational leadership in the relationship between GHRM and GIWB. To achieve these objectives, data were collected from 503 employees in hotels situated in Beijing, China, and another 498 employees in hotels in Islamabad, Pakistan. Overall, our findings provide strong support for our hypotheses, with a few noteworthy exceptions, particularly regarding the moderating role of GTL in the data collected from Islamabad, Pakistan. The results show a significant and positive relationship between GHRM and GIWB in China and Pakistan. In both datasets, the mediating role of organizational citizenship behaviour was found to be significant, emphasizing its importance in fostering environmentally responsible behaviour. Similarly, the mediation of green perceived organizational support highlighted the crucial link between employees’ perceptions and the impact of GHRM practices on GIWB. An interesting deviation in the results was observed concerning the moderating effect of GTL. In China, GTL played a positive role in enhancing the relationship between GHRM and GIWB, however, in Pakistan, GTL exhibited an insignificant moderation.

### Theoretical implications

This study contributes to the existing body of literature by extending the application of the AMO theory and organizational support theory to the context of green human resource management (GHRM) and green innovative work behavior (GIWB). By addressing the mediating and moderating mechanisms, this study advances theoretical frameworks on sustainability and organizational behavior, particularly in the underexplored context of the hospitality industry in developing and emerging economies.

### Managerial implications

The study offers practical insights for managers aiming to foster environmentally sustainable practices in the hospitality sector. These insights equip policymakers and practitioners with actionable strategies to integrate sustainability into HRM practices effectively. First, organizations should prioritize GHRM practices, such as green training, rewards, and performance appraisals, as these practices directly influence employees’ innovative behaviors towards environmental sustainability. Second, managers should actively promote policies and communication strategies that reinforce the organization’s commitment to sustainability. Furthermore, the findings highlight the need for a tailored approach to leadership. In contexts like China, GTL can significantly enhance the impact of GHRM, suggesting that leaders should be trained to adopt transformational leadership behaviors. However, in contexts like Pakistan, where the moderating role of GTL was insignificant, alternative leadership styles or strategies may be needed to achieve similar outcomes.

### Limitations and future research directions

Like any study, ours also has certain limitations. First, the generalizability of our findings may be constrained by the specific industry and geographical focus, cautioning against broad extrapolations to other sectors or regions. Additionally, the reliance on self-reported data introduces the potential for bias, as participants may have provided socially desirable responses. The study’s cross-sectional nature and lack of longitudinal data also limit our ability to capture evolving organizational and environmental dynamics over time. Future research should consider addressing these limitations, exploring diverse industries and cultures, employing objective measures, and adopting longitudinal approaches to provide a more comprehensive understanding of the relationship between GHRM, organizational behaviours, and environmental responsibility.

## Data Availability

The datasets used and/or analysed during the current study are available from the corresponding author on reasonable request.

## Appendix A

This study explores how green HRM practices influence the green innovative work behavior in the hospitality sector. Participation is voluntary, and completing the questionnaire indicates informed consent. All responses will remain confidential and anonymous, used solely for academic purposes. Thank you for your valuable input!

## **Demographics**

**Table:**

**Gender**	**Experience**
1. Male ☐ 2. Female ☐	1. 0-5 ☐ 2. 6-10 ☐ 3. 11-15 ☐ 4. 16-20 ☐ 5. 21-above ☐
**Age**	**Education**
1. 20-25 ☐ 2. 26-30 ☐ 3. 31-35 ☐ 4. 36-40 ☐ 5. 41-45 ☐ 6. 46-above ☐	1. High School ☐ 2. Bachelor’s ☐ 3. Master’s ☐ 4. Mphil/MS ☐ 5. Others ☐ Please specify (…….)

**Table:**

**Sr**	**Tick In the Relevant Box**	**Strongly Agree**	**Agree**	**Neutral**	**Disagree**	**Strongly Disagree**
	**Green Innovative Behavior**					
1.	I search out new environmentally-related technologies, processes, techniques and/or product ideas.	5	4	3	2	1
2.	I generate green creative ideas	5	4	3	2	1
3.	I promote and champion green ideas with others	5	4	3	2	1
4.	I Investigate and secure the funds needed to implement new green ideas	5	4	3	2	1
5.	I develop adequate plans and schedules for the implementation of new green ideas	5	4	3	2	1
6.	I am environmentally innovative.	5	4	3	2	1
	**Green Human Resource Management Practices**	**Strongly Agree**	**Agree**	**Neutral**	**Disagree**	**Strongly Disagree**
7.	My organization sets green goals for its employees.	5	4	3	2	1
8.	My organization provides employees with green training to promote green values.	5	4	3	2	1
9.	My organization provides employees with green training to develop employees’ knowledge and skills required for green management.	5	4	3	2	1
10.	My organization considers employees’ workplace green behavior in performance appraisals.	5	4	3	2	1
11.	My organization relates employees’ workplace green behaviors to rewards and compensation.	5	4	3	2	1
12.	My organization considers employees’ workplace green behaviors in promotion.	5	4	3	2	1
	**Organizational Citizenship Behavior Towards Environment**					
13.	In my daily activities, I weigh the environmental impact of my personal actions.	5	4	3	2	1
14.	I propose new practices that improve my facility’s environmental performance.	5	4	3	2	1
15.	I perform voluntary environmental actions and initiatives in my daily activities.	5	4	3	2	1
	**Green Perceived Organizational Support **					
16.	Our hotel values my contribution to green management issues.	5	4	3	2	1
17.	Our hotel really considers my environmental values and goals.	5	4	3	2	1
18.	Our hotel cares about my opinions on green management issues.	5	4	3	2	1
19.	Our hotel takes pride in my accomplishments on green management issues	5	4	3	2	1
20.	Our hotel would ignore any complaint from me on green management issues	5	4	3	2	1
21.	Our hotel values extra effort from me on green management issues	5	4	3	2	1
22.	Our hotel cares about my satisfaction with green management issues.	5	4	3	2	1
	**Green Transformational Leadership**					
23.	My leader inspires the organization members with the environmental plans.	5	4	3	2	1
24.	My leader provides a clear environmental vision for the members to follow.	5	4	3	2	1
25.	My leader gets the organization members to work together for the same environmental goals.	5	4	3	2	1
26.	My leader encourages the organization members to achieve the environmental goals.	5	4	3	2	1
27.	My leader acts with considering environmental beliefs of the organizational members.	5	4	3	2	1
28.	My leader stimulates the organizational members to think about green ideas.	5	4	3	2	1
